# Fungal Extracellular Vesicles are Recoverable Across Variable Ultracentrifugation Speeds but Display Species-specific Profiles of Sedimentation

**DOI:** 10.1007/s00232-026-00386-3

**Published:** 2026-06-17

**Authors:** Luisa J. Jozefowicz, Bárbara T. Bezerra, Eva Veres, Zóra Szilovics, Cássia M. Souza, Amanda C. Camillo-Andrade, Hellen G. G. Santos, Paulo C. Carvalho, Marlon D. M. Santos, Flavia C. G. Reis, Attila Gacser, Marcio L. Rodrigues

**Affiliations:** 1 Instituto Carlos Chagas, Fundação Oswaldo Cruz (Fiocruz), Curitiba, Brazil; 2 Programa de Pós-Graduação em Biologia Parasitária, Instituto Oswaldo Cruz, Fiocruz, Rio de Janeiro, Brazil; 3 Department of Biotechnology and Microbiology, University of Szeged, Szeged, Hungary; 4 Analytical Biochemistry and Proteomics Unit, IIBCE/Institut Pasteur de Montevideo, Montevideo, Uruguay; 5 HUN-REN-SZTE Pathomechanisms of Fungal Infections Research Group, University of Szeged, Szeged, Hungary; 6 Competence Centre for Molecular Biology, Bionics and Biotechnology, University of Szeged, IKIKK, Szeged, Hungary; 7 Instituto de Microbiologia Paulo de Góes (IMPG), Universidade Federal do Rio de Janeiro, Rio de Janeiro, Brazil; 8 Centre for Medical Mycology in Latin America (CMM LATAM) Unit, São Paulo, Brazil

**Keywords:** Extracellular vesicles, Fungi, Candida, Cryptococcus, Ultracentrifugation, Proteomics

## Abstract

Extracellular vesicles (EVs) are central components of fungal biology, yet their isolation commonly relies on ultracentrifugation protocols originally developed for mammalian systems. In *Cryptococcus*, most EVs carry the capsular polysaccharide glucuronoxylomannan (GXM), raising the possibility that vesicle density, and therefore sedimentation, could be influenced by polysaccharide content. Here, we systematically evaluated EV recovery across sequential ultracentrifugation speeds (20,000 × g to 100,000 × g) in *Cryptococcus deuterogattii*, *Candida auris*, and *Candida parapsilosis*. In *C. deuterogattii*, GXM was detected across all ultracentrifugation fractions, and each fraction efficiently transferred polysaccharide to acapsular cells, demonstrating that its presence is independent of fraction density and does not correlate with sedimentation behavior. In the three fungal species, transmission electron microscopy and nanoparticle tracking analysis confirmed that bona fide EVs are recoverable across all centrifugation speeds. Qualitative and quantitative proteomic analyses revealed largely overlapping protein compositions among fractions within each species. However, proteomic profiles differed between species. In *C. deuterogattii* and *C. auris*, fractions displayed similar proteomic and predicted protein-protein interaction signatures across centrifugation speeds. In contrast, *C. parapsilosis* exhibited a clear partitioning pattern, with low-speed fractions (20,000 × g and 40,000 × g) clustering together and differing from higher-speed fractions (60,000–100,000 × g). These findings demonstrate that fungal EVs are recoverable across a broad range of ultracentrifugation speeds, but their sedimentation behavior is species-specific. Our study highlights the need for tailored EV isolation strategies and cautions against assuming methodological equivalence across fungal pathogens.

**Graphical Abstract:**

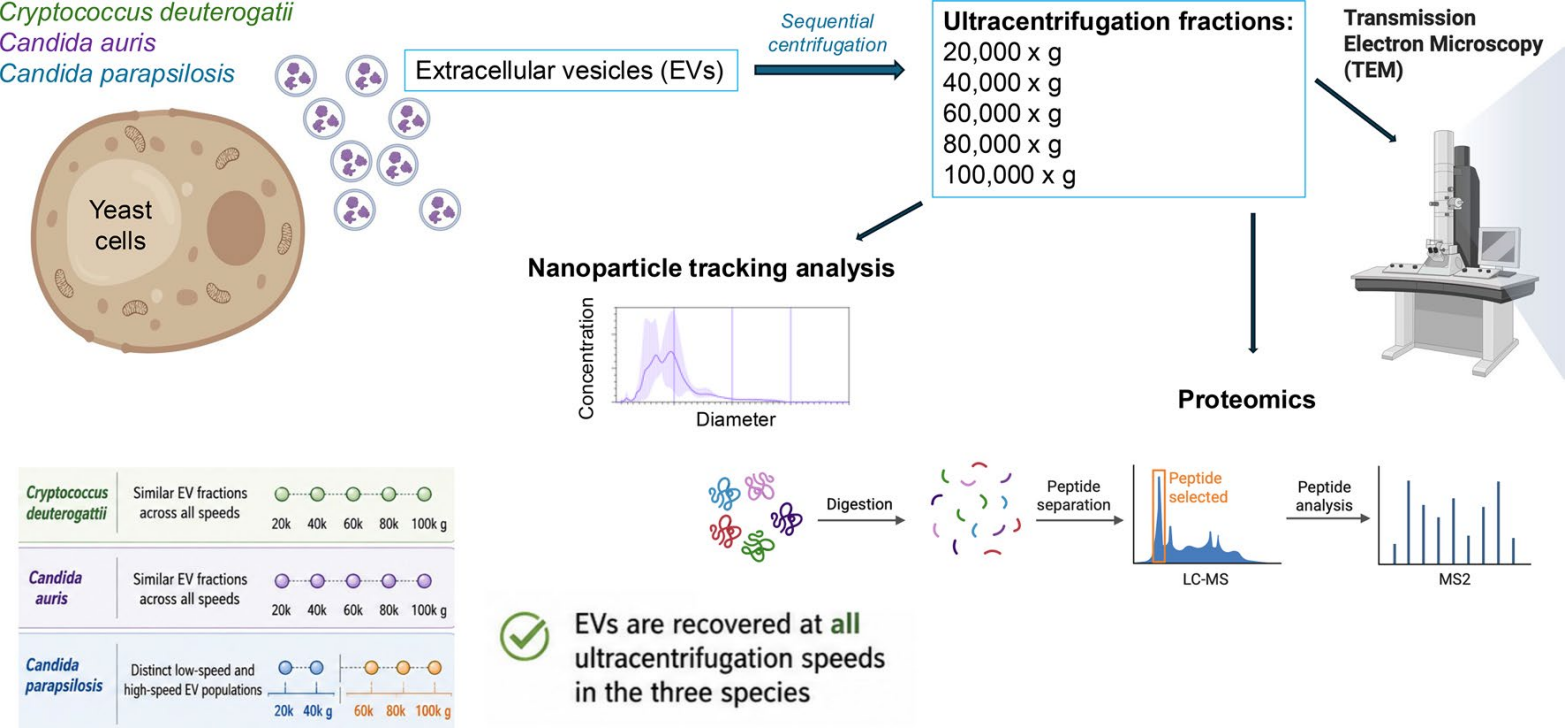

**Supplementary Information:**

The online version contains supplementary material available at 10.1007/s00232-026-00386-3.

## Key Message

Extracellular vesicles (EVs) are central to fungal biology, but their isolation still relies on protocols adapted from mammalian systems, with limited exploration of more appropriate protocols for fungi. Here, we show that EVs from major fungal pathogens are recovered across a broad range of ultracentrifugation speeds, and that their sedimentation behavior is species-specific. These findings challenge the use of fixed ultracentrifugation conditions as a universal standard and highlight the need to tailor EV isolation strategies for different fungal species, with important implications for reproducibility and data interpretation in the field.

**Supplementary Information:**

The online version contains supplementary material available at 10.1007/s00232-026-00386-3.

## Introduction

Fungal diseases kill millions of individuals each year (Denning 2024). Despite their major public health impact, no licensed fungal vaccines are currently available, and antifungal therapy is compromised by drug resistance, toxicity, and limited access to appropriate treatments in neglected populations (Rodrigues and Nosanchuk 2020). The development of new strategies to combat fungal infections requires a deeper understanding of fungal biology, a field that has historically received less attention than other infectious diseases of comparable importance (Rodrigues and Albuquerque 2018).

Extracellular vesicles (EVs) have emerged as key components of fungal biology that directly influence pathogenic potential, thereby representing promising targets for drug and vaccine development (Rodrigues et al. 2025). However, a major limitation in the fungal EV field is the lack of methodological approaches designed to separate vesicle subpopulations and associate them with specific biological functions. As a result, important aspects of fungal EV biogenesis, reliable biomarkers, and the identification of bioactive subpopulations remain insufficiently understood (Rodrigues et al. 2025).

Fungal EVs were initially characterized using ultracentrifugation-based protocols adapted from mammalian EV research (Rodrigues et al. 2007). This approach appeared reasonable, as fungal EVs share several features with mammalian vesicles, supporting their classification into categories such as exosomes and microvesicles (Rodrigues et al. 2025). However, it has become increasingly evident that fungal EVs possess unique properties. For example, they can be readily isolated from cultures grown on solid media (Reis et al. 2019), suggesting that they are produced under highly diverse conditions. Moreover, fungal EV biogenesis can involve mechanisms distinct from those described in mammalian cells, including plasma membrane remodeling events that release cytoplasmic portions in ways not observed in mammalian systems (Rodrigues et al. 2013). Given these differences, it is plausible that protocols optimized for mammalian EV isolation may not be ideal for fungal vesicles. Conversely, alternative methodological approaches may uncover previously unrecognized features of fungal EV biology.

A distinctive characteristic of fungal EVs is their ability to transport polysaccharides extracellularly (Rodrigues et al. 2007; da Silva et al. 2015). In *Cryptococcus*, a major human pathogen, the principal virulence factor, the capsular polysaccharide glucuronoxylomannan (GXM), is exported in EVs and can be incorporated into the fungal surface to build the polysaccharide capsule (Rodrigues et al. 2007). Because GXM and other polysaccharides are highly viscous and dense (Frases et al. 2008), vesicle density, and consequently sedimentation behavior during ultracentrifugation, could theoretically be influenced by polysaccharide content.

In this study, we tested whether GXM-containing *Cryptococcus* EVs could be preferentially recovered at lower ultracentrifugation speeds due to increased density. Contrary to our hypothesis, GXM was similarly distributed among fractions sequentially recovered across ultracentrifugation speeds ranging from 20,000 × g to 100,000 × g. We further demonstrated that bona fide EVs can be recovered across this entire range of centrifugal forces. We extended these observations to two additional pathogens, *Candida auris* and *Candida parapsilosis*, which have no described ability to export polysaccharides in EVs. Proteomic analyses revealed broad similarities among fractions within each species, but also uncovered species-specific sedimentation profiles. Together, these findings reveal that fungal EVs are recoverable across a wide range of ultracentrifugation speeds while displaying species-dependent sedimentation behavior. Our results provide new insight into fungal EV biology and underscore the importance of species-specific methodological optimization in vesicle research.

## Results

### Cryptococcal GXM is Distributed among Ultracentrifugation Fractions Obtained at Different Speeds

**Fig. 1:**
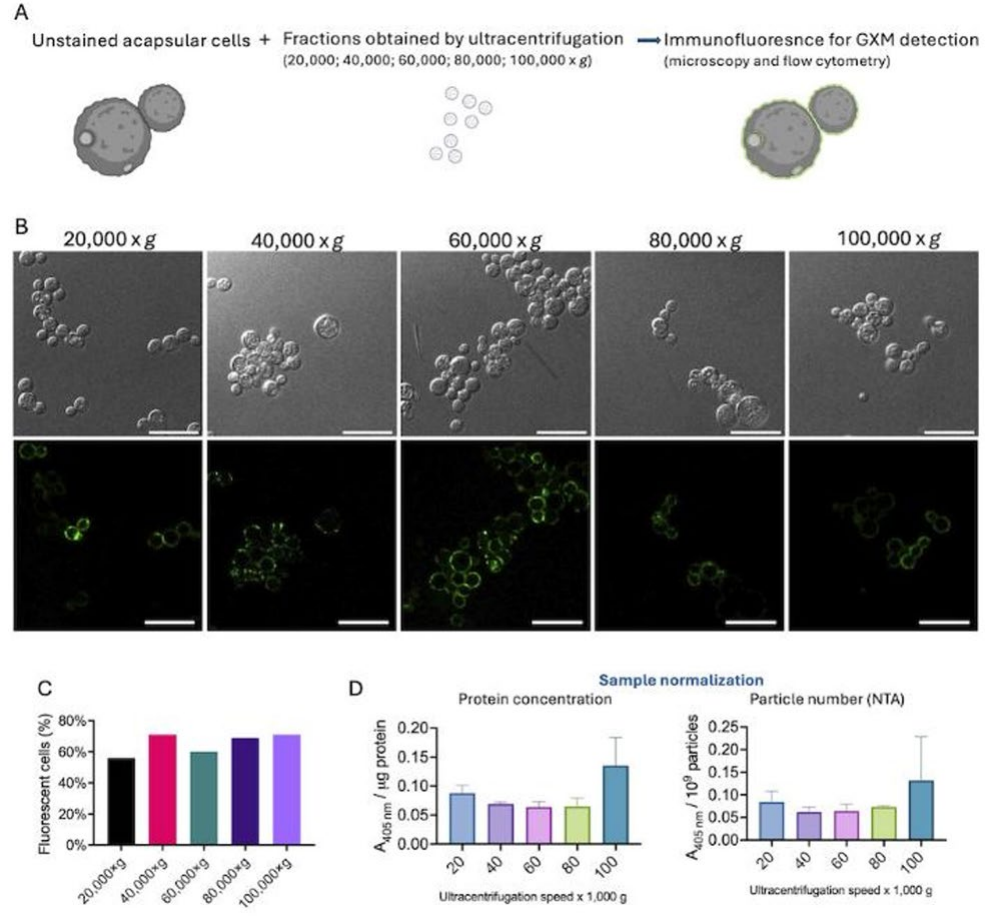
GXM detection in *C. deuterogattii* fractions obtained by sequential ultracentrifugation at 20,000, 40,000, 60,000, 80,000, and 100,000 × g. (**A**) Schematic representation of the experimental design used to test the ability of each fraction to serve as a source of GXM for an acapsular mutant of *C. neoformans*. (**B**) Representative analysis of GXM incorporation into the surface of acapsular cells after incubation with EV fractions recovered at the indicated centrifugation speeds. Upper panels represent yeast cells incubated with each fraction under differential interferential contrast (scale bars, 200 nm). Lower panels represent yeast cells stained with a monoclonal antibody raised to GXM (green fluorescence). (**C**) Quantification of GXM-positive acapsular cells by flow cytometry following incubation with each fraction. (**D**) GXM quantification in each EV fraction by ELISA using two normalization methods. Comparable polysaccharide levels were detected across all ultracentrifugation fractions, as determined by ANOVA (*p* = 0.61 for protein concentration and *p* = 0.3 for particle number normalization)

Cryptococcal GXM is exported in EVs (Rodrigues et al. 2007), and the majority of vesicles produced by *Cryptococcus* (approximately 70%) give positive results for GXM detection (Rizzo et al. 2021). Because GXM is highly viscous and dense (Frases et al. 2008), we hypothesized that EVs containing this polysaccharide might pellet at lower ultracentrifugation speeds. To test this possibility, we applied a sequential ultracentrifugation protocol to separate denser EVs from lighter particles. The standard strain R265 of *C. deuterogattii* was selected as a model over better studied cryptococcal strains due to its higher EV production compared to *C. neoformans* (Reis et al. 2021). Culture supernatants from *C. deuterogattii* (strain R265), cleared of cells and debris, were first ultracentrifuged at 20,000 × g. The pellet was collected and stored at 4 °C, and the supernatant was subsequently centrifuged at 40,000 × g. This process was repeated sequentially at 60,000, 80,000, and 100,000 × g, generating five fractions (pellets obtained at 20,000, 40,000, 60,000, 80,000, and 100,000 × g). Given that acapsular cryptococci can extract GXM from EVs and incorporate it into their surface (Rodrigues et al. 2007), we evaluated each fraction for its ability to serve as a GXM source for an acapsular mutant of *C. deneoformans* (Fig. 1A). All fractions behaved similarly (Fig. 1B), supporting efficient GXM incorporation into the surface of *C. neoformans*. Flow cytometry analysis showed that approximately 60% of acapsular cells became GXM-positive after incubation with each ultracentrifugation fraction, regardless of the centrifugation speed (Fig. 1C). We used ELISA with an antibody to GXM to quantify the polysaccharide in each sample (Casadevall et al. 1992). If GXM were a major determinant of vesicle density, we would expect its enrichment in fractions recovered at lower centrifugation speeds, which are typically enriched in larger and/or more dense particles that sediment more readily. To test this, we normalized GXM content using two independent approaches - total protein concentration and particle number determined by NTA for each fraction. Despite variations in normalized GXM content among independent experiments, particularly in the fraction obtained at 100,000 x g, no statistically significant differences were observed among fractions obtained at the various ultracentrifugation speeds (ANOVA, *p* = 0.61 for protein concentration and *p* = 0.3 for particle number normalization). Together, these results indicate that GXM abundance does not correlate with sedimentation behavior and is unlikely to be a primary driver of vesicle density.

### EVs Produced by Three Fungal Species are Recoverable across a Range of Ultracentrifugation Speeds

**Fig. 2:**
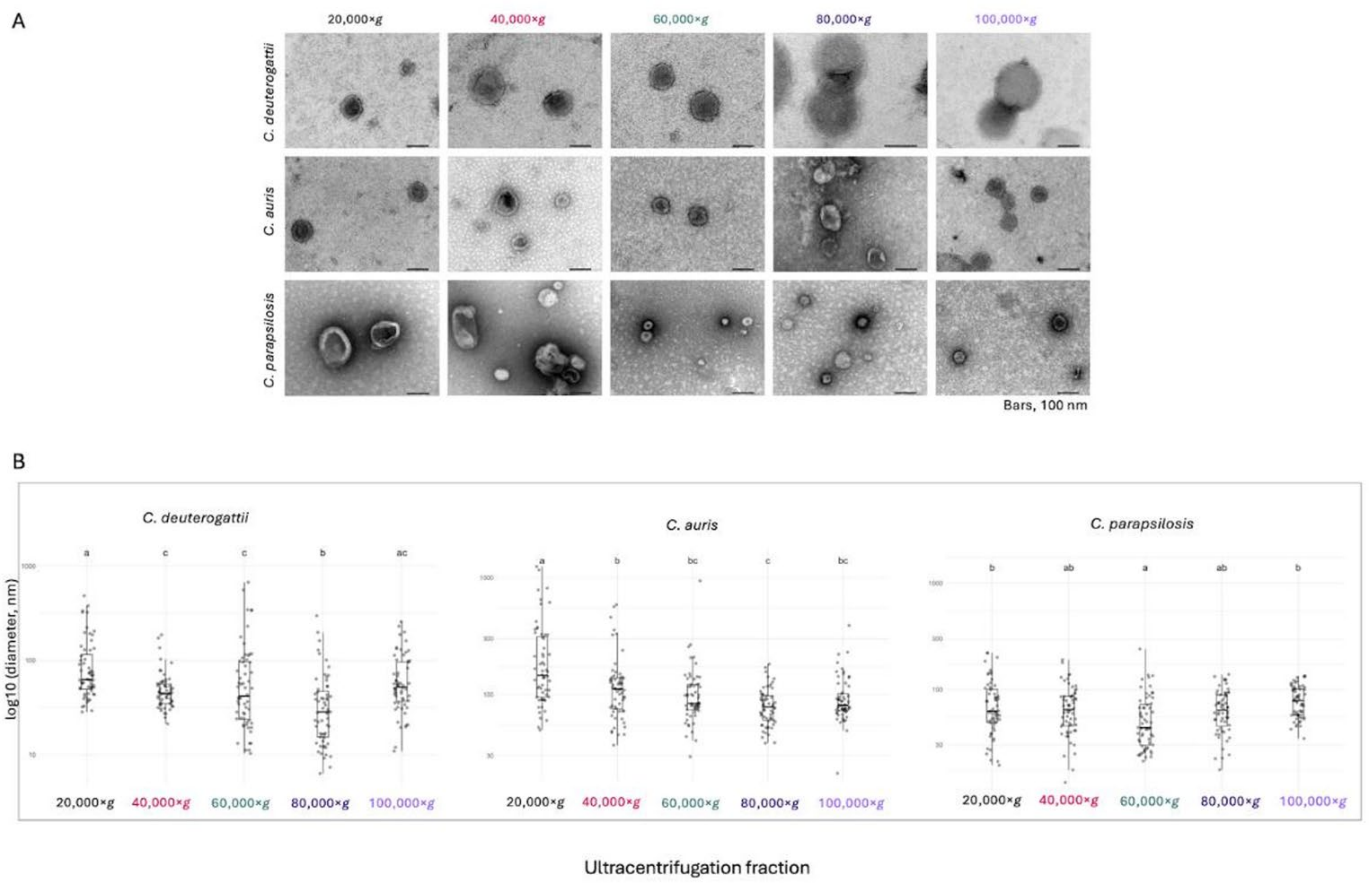
Fungal EVs are recoverable across variable ultracentrifugation speeds. (**A**) TEM images of EV pellets obtained by sequential ultracentrifugation at 20,000, 40,000, 60,000, 80,000, and 100,000 × g from *C. deuterogattii* (first lane), *C. auris* (second lane), and *C. parapsilosis* (third lane). All fractions exhibited the typical morphological features of fungal EVs. (**B**) Vesicle diameter measurements for each ultracentrifugation fraction. Size distribution analysis revealed variable dimensions across different speeds for all three species. In this analysis, groups that share the same letter do not exhibit statistically significant differences, as determined by Dunn’s post hoc test with Holm adjustment. A tendency toward larger vesicles was observed in the 20,000 × g fraction of *C. auris*, whereas no consistent size shift was detected in *C. deuterogattii* or *C. parapsilosis*

The absence of an effect of GXM on the sedimentation profile of cryptococcal EVs led us to question whether the fractions obtained by sequential ultracentrifugation indeed corresponded to bona fide EVs. We also asked whether EVs could be recovered at different ultracentrifugation speeds in fungi that do not produce GXM. Transmission electron microscopy (TEM) of each cryptococcal fraction (Fig. 2A, first lane) showed that pellets obtained at 20,000, 40,000, 60,000, 80,000, and 100,000 × g shared the typical morphological features of fungal EVs, including a round shape and bilayered membranes. Comparable results were observed for the GXM non-producing fungi *Candida auris* and *Candida parapsilosis* (Fig. 2A, second and third lanes). These findings indicated that fungal EVs can be pelleted across a broad range of ultracentrifugation speeds. To explore the basis of this observation, we asked whether larger vesicles would preferentially sediment at lower centrifugation speeds. We therefore measured vesicle diameters in each fraction (Fig. 2B). Differences in individual-particle vesicle diameter distributions among fractions obtained at different ultracentrifugation speeds varied according to the species. In *C. auris*, vesicles from the 20,000 × g fraction had larger diameters than those from all other fractions. However, this pattern was not observed for *C. deuterogattii* or *C. parapsilosis*. In *C. deuterogattii*, vesicles from the 80,000 × g fraction had the smallest diameters and differed from those obtained at all other speeds. In *C. parapsilosis*, significant differences were detected for vesicles from the 60,000 × g fraction, which had smaller diameters than those from the 20,000 × g and 100,000 × g fractions (Dunn’s post hoc test with Holm’s adjustment, *p* < 0.05).

### EV Fractions Recovered at Different Ultracentrifugation Speeds Share Similar Properties

**Fig. 3:**
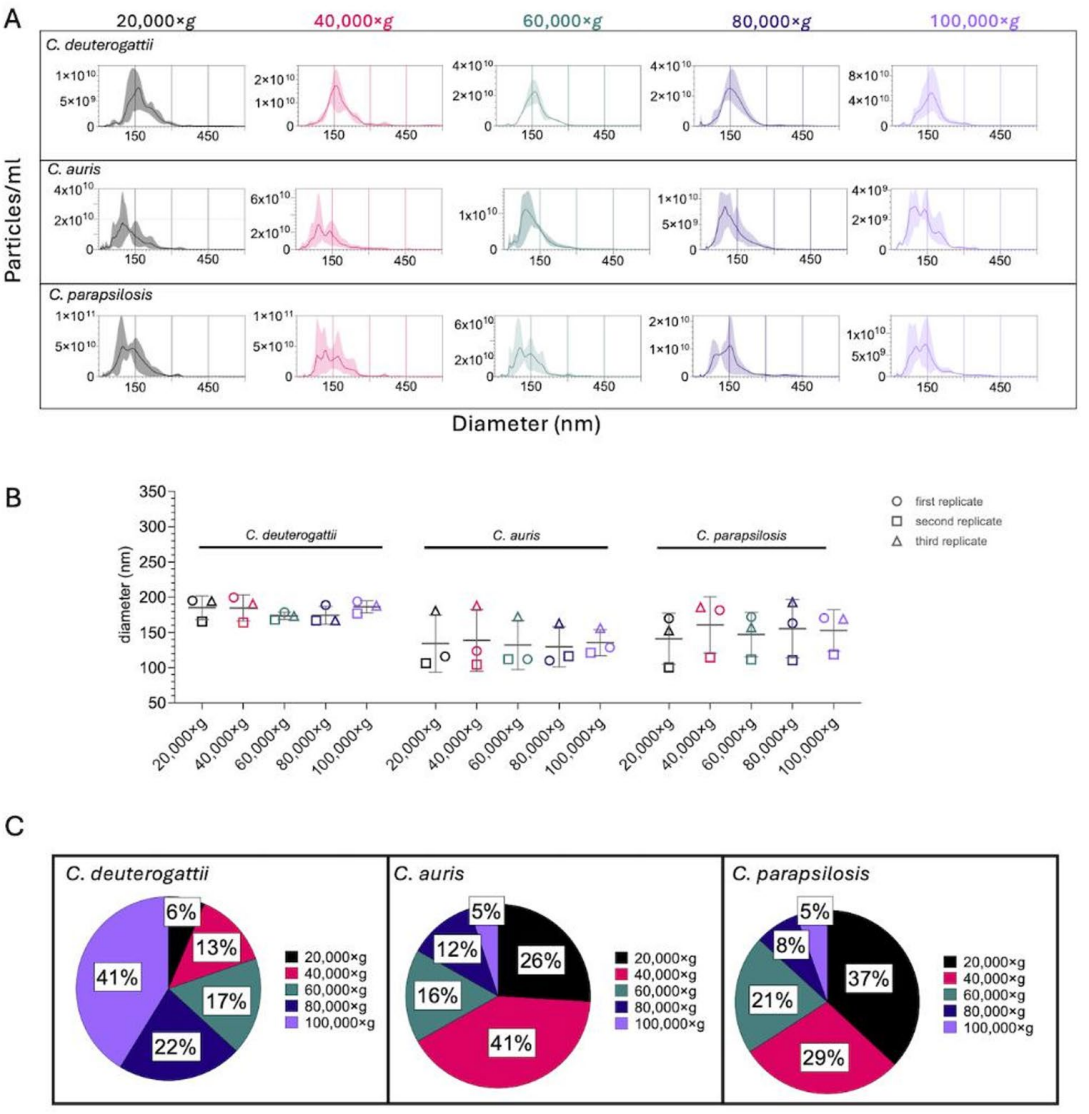
EV fractions recovered at different ultracentrifugation speeds share similar NTA distributions but display species-specific recovery patterns. (A) Representative NTA size distribution profiles for each ultracentrifugation fraction from *C. deuterogattii*,* C. auris* and *C. parapsilosis*. (B) Mean vesicle diameters for each fraction. No statistically significant differences in particle size were observed among fractions within each species (*C. auris*, *p* = 0.88; *C. deuterogattii*, *p* = 0.16; *C. parapsilosis*, *p* = 0.27). C. Distribution of total particle recovery across ultracentrifugation fractions for each species

Vesicle diameter was also measured under automated conditions by nanoparticle tracking analysis (NTA) in EV fractions from *C. deuterogattii*, *C. auris*, and *C. parapsilosis* obtained by sequential ultracentrifugation. Consistent with the TEM results, all fractions displayed the typical NTA size distribution reported for fungal EVs, with most particles ranging from 100 to 150 nm (Fig. 3A). Based on the mean diameters obtained by automatic measurements (Fig. 3B), no significant differences among ultracentrifugation speeds were observed in any of the three species (*C. auris*, *p* = 0.88; *C. deuterogattii*, *p* = 0.16; *C. parapsilosis*, *p* = 0.27). These findings support the absence of a correlation between vesicle size and sedimentation at different ultracentrifugation speeds, regardless of the fungal species analyzed. In contrast, EV concentration varied markedly among fractions and species (Fig. 3C). Cryptococcal EVs were predominantly recovered at higher speeds, with 41% and 22% of particles detected in the 100,000 × g and 80,000 × g fractions, respectively. In comparison, EVs from *Candida* species were enriched in lower-speed fractions. In *C. auris*, the 20,000 × g and 40,000 × g fractions accounted for 26% and 41% of total particles, respectively, whereas in *C. parapsilosis* these fractions contained 37% and 29% of particles. Together, these results indicate that although fungal EVs can be recovered across a range of ultracentrifugation conditions, the efficiency of particle recovery at specific speeds depends on the species under investigation. Of note, EV densities were not measured, and all comparisons described above are diameter-centered, which included the typically described discrepancies between NTA versus TEM measurements (van der Pol et al. 2014). This is likely explained by the well-reported efficacy of TEM to detect the smallest vesicles, in contrast to NTA (van der Pol et al. 2014).

### Proteomic Analysis of EV Fractions from *C. deuterogattii*,* C auris*, and *C parapsilosis*

**Fig. 4:**
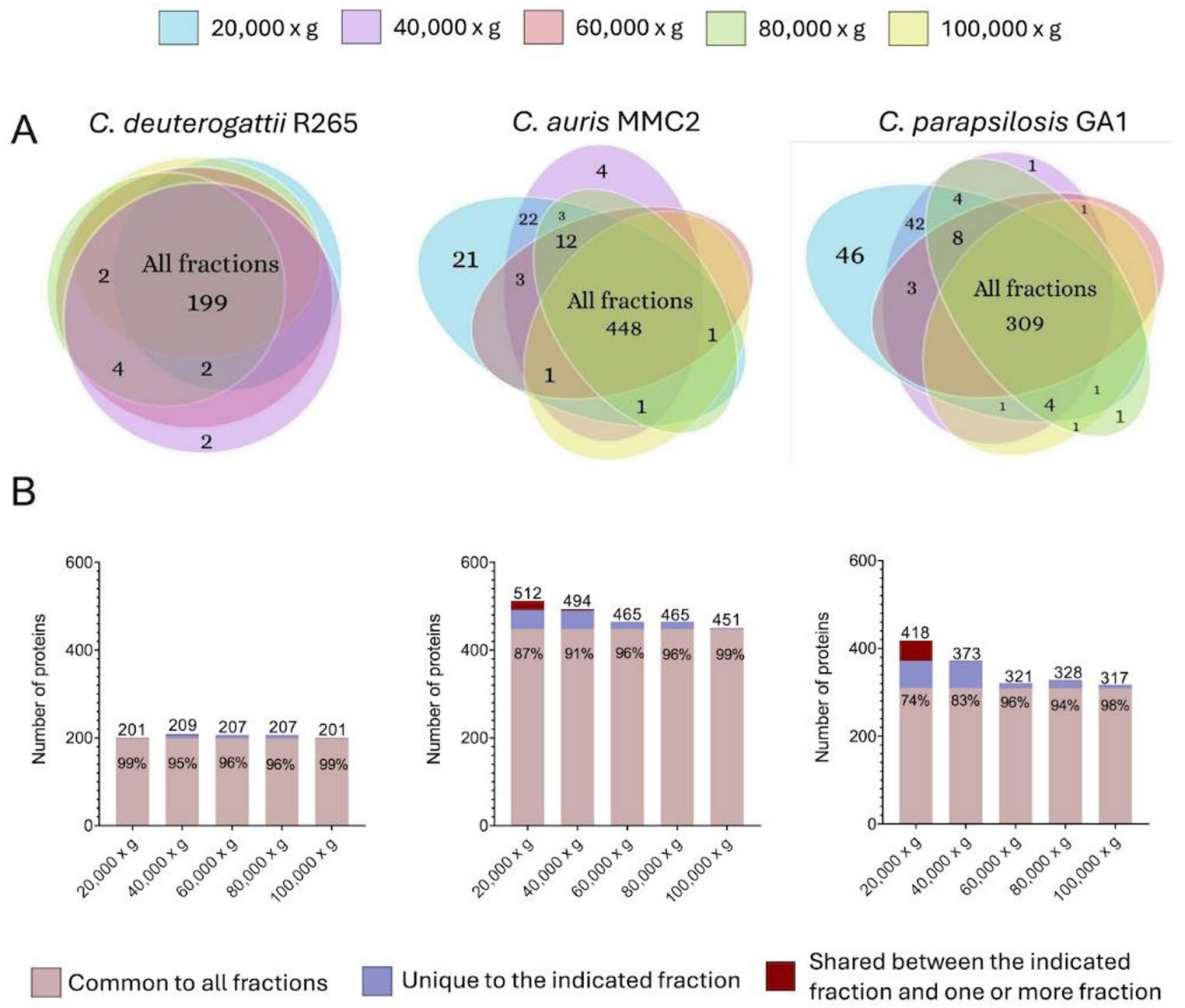
Qualitative proteomic analysis of EV fractions obtained at different ultracentrifugation speeds. Analysis of cryptococcal proteins revealed a similar distribution pattern across all fractions, as indicated by the predominance of shared proteins in the Venn diagrams (A). Most proteins were shared among all fractions (B). A predominance of shared proteins was also observed in *C. auris* (C), although the detection of fraction-specific proteins (D) was higher than in *C. deuterogattii*. Similar results were observed in the Venn diagrams for *C. parapsilosis* (E) and in its protein distribution across fractions (F). Only proteins detected in at least two replicates were included in this analysis. The diagrams show proteins uniquely identified in each specified fraction as well as those shared among one or more fractions. The percentage of common proteins in each fraction is also shown in each column

We next investigated whether the protein composition of fungal EVs varied among the fractions obtained for the three species analyzed. To address this question, the 20,000, 40,000, 60,000, 80,000, and 100,000 × g fractions were subjected to proteomic analysis. The complete protein datasets for each fraction are provided in Supplemental Files 1 (*C. deuterogattii*), 2 (*C. auris*), and 3 (*C. parapsilosis*). For each species, we first determined which proteins were shared across all fractions. In *C. deuterogattii*, the number of identified proteins was highly similar among fractions, ranging from 201 to 207 (95 to 99%). Notably, 199 proteins were common to all five fractions (Fig. 4A). Accordingly, the overall protein composition was highly conserved, with more than 95% overlap among fractions (Fig. 4B). In *C. auris*, the total number of proteins per fraction ranged from 451 to 512 (Fig. 4C). A total of 448 proteins were shared across all fractions. In this species, the number of detected proteins decreased progressively with increasing ultracentrifugation speed (Fig. 4D), consistent with the higher EV concentration observed in low-speed fractions. Although most proteins were common to all fractions (87 to 99%), *C. auris* exhibited greater inter-fraction variability than *C. deuterogattii*, including a higher number of fraction-specific proteins, particularly in the 20,000 × g fraction (21 proteins). The greatest inter-fraction variability was observed in *C. parapsilosis*. In this species, protein numbers ranged from 317 to 418 (74 to 98%), with 309 proteins shared among all fractions (Fig. 4E). As observed for *C. auris*, the number of proteins per fraction tended to decrease with increasing centrifugation speed. Notably, the highest number of exclusive proteins was detected in the 20,000 × g fraction of *C. parapsilosis* (46 proteins). In summary, EV fractions obtained at different ultracentrifugation speeds displayed largely overlapping protein compositions. However, important differences were observed, and these were more pronounced in *Candida* species than in *C. deuterogattii*.

**Fig. 5:**
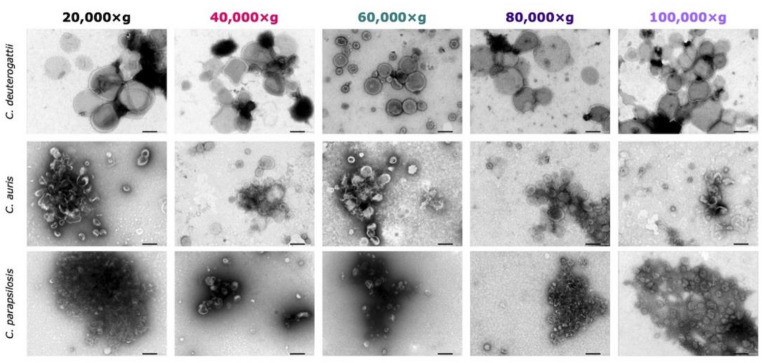
TEM reveals widespread and heterogeneous EV aggregation across centrifugation fractions. Representative transmission electron microscopy images of EVs isolated from *C. deuterogattii*, *C. auris*, and *C. parapsilosis* following sequential ultracentrifugation at 20,000 × g, 40,000 × g, 60,000 × g, 80,000 × g, and 100,000 × g. EV aggregates were detected in all fractions and across all species. Aggregate distribution appeared stochastic, with no consistent enrichment at specific centrifugation speeds. In addition, aggregates displayed marked heterogeneity in size and organization, with substantial variation in the number of vesicles per aggregate

Aggregate formation provides a plausible explanation for the apparent paradox of similar protein composition across fractions despite differential sedimentation behavior. Larger aggregates composed of comparable vesicular subunits would be expected to pellet at lower centrifugation speeds, whereas smaller or less complex assemblies would require higher speeds, thereby generating distinct sedimentation profiles without substantial changes in proteomic composition. Therefore, we searched for EV aggregates in all centrifugation fractions produced by the three fungi. EV aggregates were observed in all centrifugation fractions across the three fungal species (Fig. 5). We attempted to quantify these structures; however, this was not feasible due to their pronounced heterogeneity and apparent stochastic distribution, reflected by the lack of consistent enrichment in specific fractions and the wide variation in the number of vesicles per aggregate. Together, these features precluded the identification of reproducible patterns or fraction-dependent trends, suggesting the absence of clearly defined parameters governing aggregate formation under the conditions tested.

**Fig. 6:**
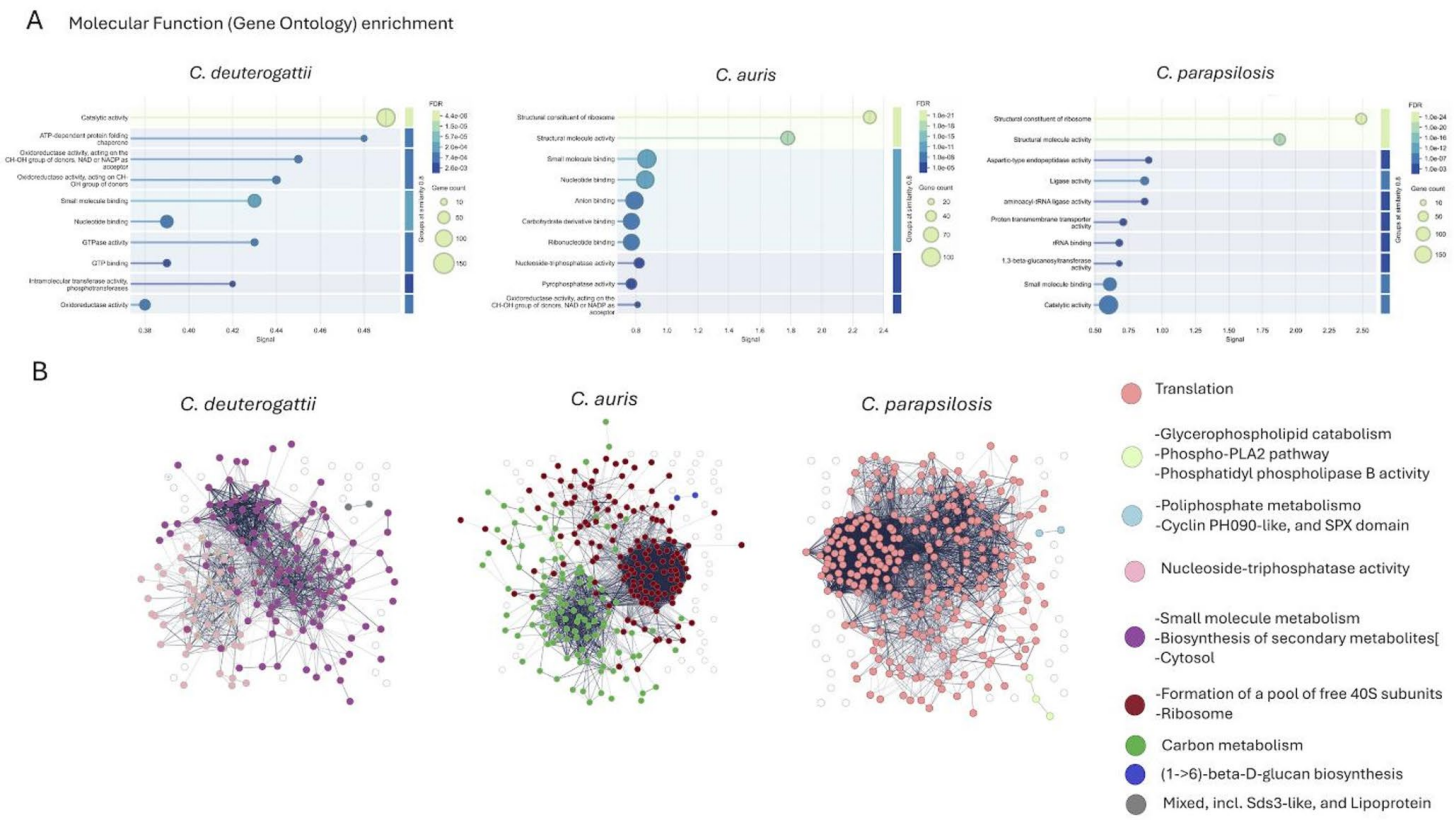
Species-specific functional and interaction profiles of core EV proteins. (**A**) Enrichment analysis of molecular functions based on proteins shared across all five centrifugation fractions in each fungal pathogen, which were considered representative of core EV-associated components. The predominant functional categories differed among species, with *C. auris* and *C. parapsilosis* showing more similar profiles, whereas *C. deuterogattii* displayed a more distinct distribution. (**B**) Predicted protein–protein interaction networks generated using STRING from the same set of shared proteins. Each species exhibited a markedly different interaction network, indicating divergence in the organization and potential functional relationships of EV-associated proteins. Molecular categories are specified for all systems

The proteomic analysis of centrifugation fractions obtained from the three fungal pathogens suggested interspecies variation in EV protein composition. To further assess this possibility, we selected the proteins shared across all five centrifugation fractions within each pathogen, and considered them the most representative EV-associated components. We used this dataset for interspecies comparisons, and first evaluated the enrichment of molecular functions in each species. The predominant categories (Fig. 6A) differed across pathogens, with *C. auris* and *C. parapsilosis* displaying greater similarity to each other, while *C. deuterogattii* exhibited a more distinct profile. Analysis of predicted protein–protein interactions using the same protein set in STRING (Fig. 6B) revealed markedly different interaction networks among the three pathogens. Together, these findings indicate that, despite some similarities in protein distribution across fractions obtained by sequential ultracentrifugation, the biological identity of EVs is strongly species-specific, as concluded from the analysis of the vesicular proteomes of these pathogens.

**Fig. 7:**
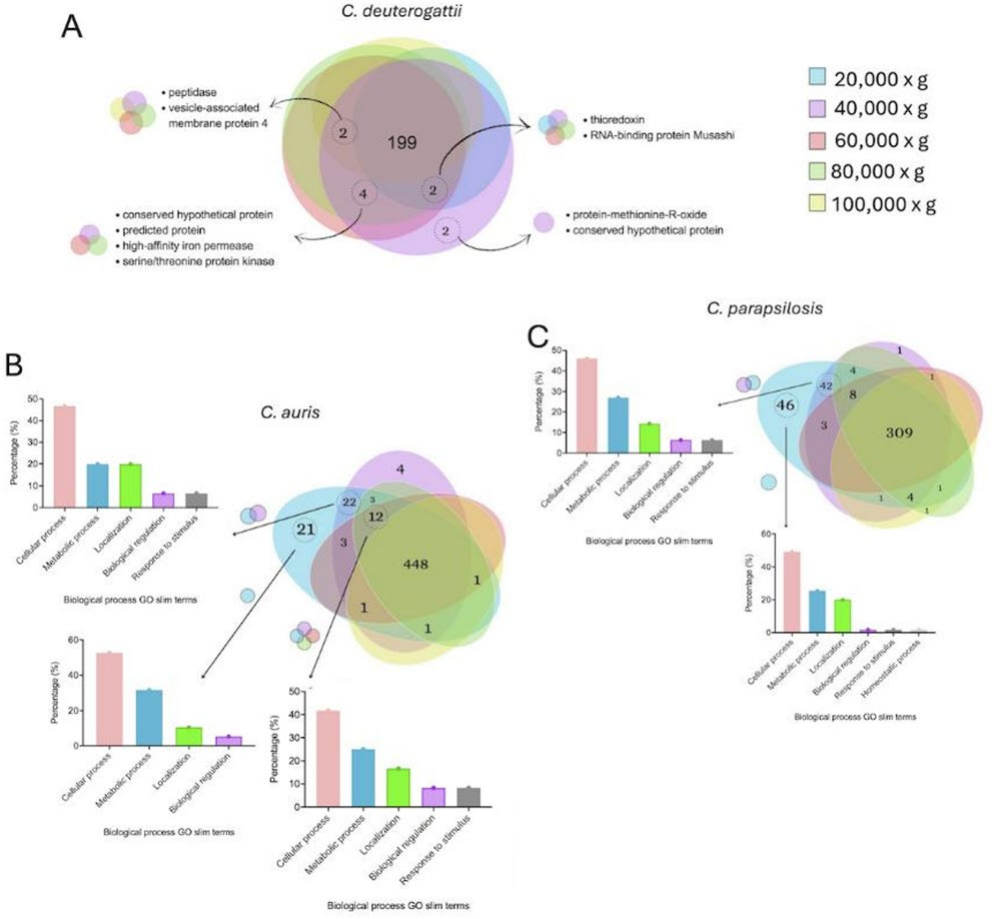
Functional characterization of fraction-specific proteins in EV preparations. (**A**) In *C. deuterogattii*, the limited number of fraction-specific proteins precluded broad functional classification. Individual analysis revealed selective enrichment or absence of specific proteins across fractions. (**B**) In C. *auris*, low-speed fractions (20,000 and 40,000 × g) were enriched in proteins associated with response to stimulus, localization, biological regulation, cellular and metabolic processes. C. In *C. parapsilosis*, the 20,000 and 40,000 × g fractions contained exclusive proteins linked to cellular processes, localization, metabolic processes, response to stimulus, and homeostatic functions. The 20,000 × g fraction showed the most prominent enrichment in these categories

The subtle compositional variations observed among fractions further substantiate the notion that differential centrifugation lacks the ability to distinguish between EV populations. To further validate or refute this hypothesis, we explored whether these subtle differences could be attributed to fraction-specific biological functions. To address this, we analyzed the cellular processes associated with the sets of exclusive proteins identified in each fraction. In *C. deuterogattii*, the small number of fraction-specific proteins did not allow a meaningful classification based on biological process. Therefore, we examined these proteins individually (Fig. 7A). Among those not universally detected, a peptidase and a vesicle-associated membrane protein were present in all samples except the 20,000 × g fraction. Conversely, thioredoxin and an RNA-binding protein were detected in all samples except the 100,000 × g fraction. Two proteins of unknown function, together with a high-affinity iron permease and a serine/threonine protein kinase, were exclusively identified in the 40,000, 60,000, and 80,000 × g fractions. In addition, a hypothetical protein and a protein-methionine-R-oxide reductase were detected only in the 40,000 × g fraction.

In *C. auris* (Fig. 7B), exclusive protein sets containing at least 10 proteins were classified according to biological process. Twelve proteins associated with cellular process, localization, metabolic processes, biological regulation and response to stimulus were identified in the 20,000, 40,000, 60,000, and 80,000 × g fractions. The 20,000 and 40,000 × g overlap and the 20,000 × g fraction also included proteins related to the same biological process GO terms but with different distributions. In *C. parapsilosis* (Fig. 7C), the 20,000 and 40,000 × g fractions exclusively contained proteins associated with cellular processes, localization, metabolic processes, response to stimulus, and homeostatic processes. The 20,000 × g fraction, in particular, included exclusive proteins linked to cellular and metabolic processes, localization, biological regulation, and response to stimulus. Taken together, these findings further highlight similarities among *Candida* EV fractions and point to biological processes enriched in low-speed ultracentrifugation fractions. The complete lists of proteins exclusive to each fungal fraction are provided in Supplemental Files 4 (*C. deuterogattii*), 5 (*C. auris*), and 6 (*C. parapsilosis*).

### Quantitative Proteomic Analysis of EV Fractions from *C. deuterogattii*, *C. auris*, and *C. parapsilosis*

**Fig. 8:**
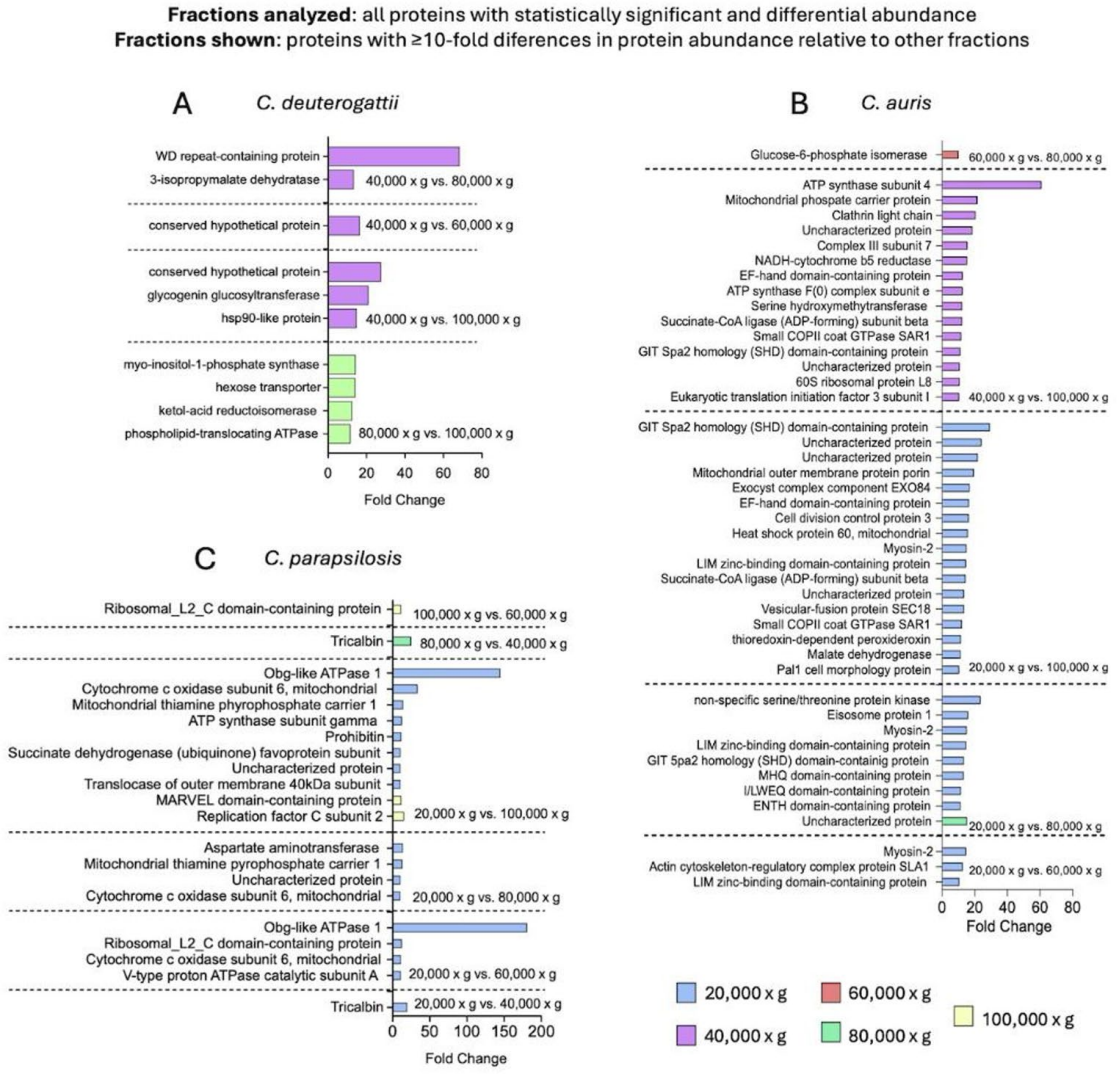
Quantitative proteomic analysis of EV fractions obtained at distinct ultracentrifugation speeds. EV fractions (20,000, 40,000, 60,000, 80,000, and 100,000 × g) from *C. deuterogattii*, *C. auris*, and *C. parapsilosis* were compared within each species. Proteins with statistically significant differential abundance (meeting both fold change and q-value criteria) and displaying at least a 10-fold difference in abundance relative to other fractions were selected for analysis

To determine whether sequential ultracentrifugation generated quantitative differences in protein abundance among EV fractions, we compared all fractions within each species and selected proteins showing at least a 10-fold difference in abundance relative to other fractions (Fig. 8). In *C. deuterogattii*, only a few proteins met this threshold. Enrichment in the 80,000 × g fraction relative to the 100,000 × g fraction included functionally unrelated proteins such as myo-inositol-phosphate synthase, a hexose transporter, keto-acid reductoisomerase, and a phospholipid-translocating ATPase. Comparison of the 40,000 × g and 100,000 × g fractions revealed higher levels of a conserved hypothetical protein, a glycogenin glucosyltransferase, and an Hsp90-like protein in the 40,000 × g fraction. Additional differences included a conserved hypothetical protein enriched in the 40,000 × g fraction compared with the 60,000 × g fraction, as well as 3-isopropylmalate dehydratase and a WD repeat-containing protein enriched in the 40,000 × g fraction relative to the 80,000 × g fraction. Overall, the small number of differentially abundant proteins supports the strong quantitative similarity among *C. deuterogattii* fractions.

In *C. auris*, more pronounced differences were observed. The 20,000 × g fraction, compared with the 60,000 × g fraction, was enriched in myosin-2, a cytoskeleton regulatory complex protein, and a zinc-binding domain protein. Comparison between the 20,000 × g and 80,000 × g fractions showed enrichment of nine proteins, particularly a non-specific serine/threonine protein kinase. The 60,000 × g fraction, compared with the 80,000 × g fraction, was enriched in glucose-6-phosphate isomerase. More substantial differences were observed in the comparison between the 20,000 × g and 100,000 × g fractions, which revealed 17 proteins highly abundant in the former. Similarly, comparison between the 40,000 × g and 100,000 × g fractions revealed 15 proteins more abundant in the 40,000 × g fraction. These findings agree with the qualitative analysis, in which the low-speed fractions (20,000 × g and 40,000 × g) showed the most marked differences. In *C. parapsilosis*, the 20,000 × g fraction, compared with the 40,000 × g fraction, was enriched in tricalbin. Similar results were obtained in the comparison between the 80,000 × g and 40,000 × g fractions. Comparison between the 20,000 × g and 60,000 × g fractions revealed highly abundant Obg-like ATPase 1, in addition to three other proteins enriched in the 20,000 × g fraction. The same protein was also highly enriched in the comparison between the 20,000 × g and 100,000 × g fractions. The 20,000 × g fraction, compared with the 80,000 × g fraction, was similarly enriched in four unrelated proteins. Finally, the 100,000 × g fraction, compared with the 60,000 × g fraction, was enriched in a ribosomal L2 C-domain-containing protein. Together, these analyses indicate that EV fractions obtained at different ultracentrifugation speeds are largely comparable within each species, but the extent of fraction-dependent enrichment varies among species and is more pronounced in *Candida* than in *C. deuterogattii*.

### Protein Interaction Network Analysis Reveals Species-dependent Fraction Clustering Patterns

**Fig. 9:**
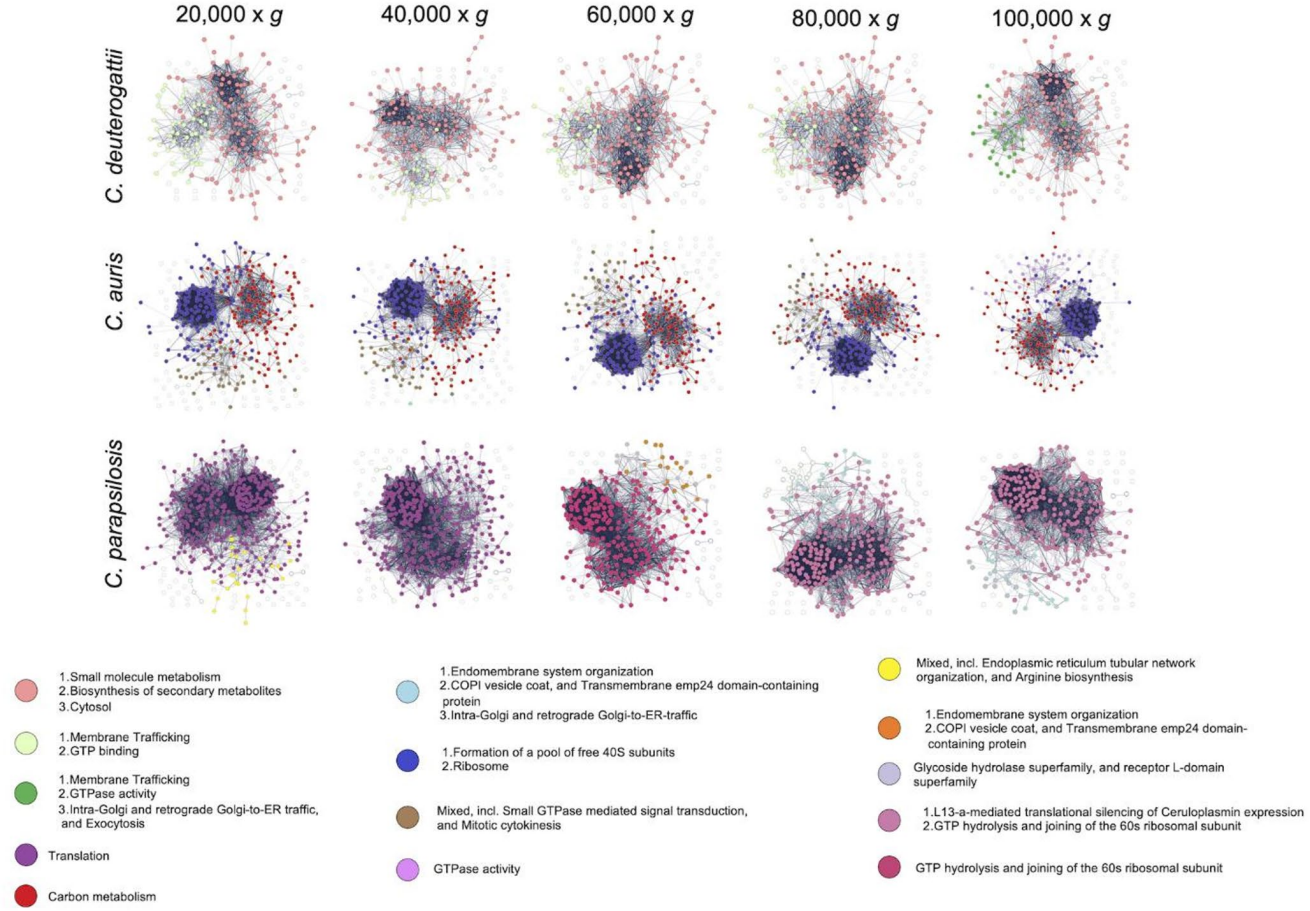
Protein interaction network analysis reveals species-dependent clustering patterns among EV fractions. Predicted protein–protein interaction networks were reconstructed for EV fractions (20,000, 40,000, 60,000, 80,000, and 100,000 × g) from *C. deuterogattii*, *C. auris*, and *C. parapsilosis* using STRING-based analysis. Proteins were grouped into five functional clusters based on k-means clustering analysis using the STRING database. Clusters are color-coded and labeled according to the original functional categories provided by STRING. Only clusters with at least 10 proteins are shown. In *C. deuterogattii*, interaction networks derived from all five fractions displayed highly similar topological organization and functional clustering. A comparable pattern was detected in *C. auris*. *C. parapsilosis* exhibited a distinct fraction-dependent pattern. The 20,000 × g and 40,000 × g fractions generated highly similar networks characterized by functional clusters primarily associated with translational processes. The 60,000 × g, 80,000 × g, and 100,000 × g fractions displayed networks with distinct organizational profiles, indicating a shift in predicted interaction signatures at higher centrifugation speeds

To investigate whether fraction-dependent differences were reflected at the level of predicted protein–protein interactions, we analyzed each EV fraction using STRING-based network reconstruction (Fig. 9). In *C. deuterogattii*, the interaction networks generated for the 20,000 × g, 40,000 × g, 60,000 × g, 80,000 × g, and 100,000 × g fractions displayed highly similar topological organization and functional clustering patterns. The same major functional modules were observed across all fractions, with a predominance of interactions related to small molecule metabolism, biosynthesis of secondary metabolites and cytosolic processes. Although an additional cluster was observed in the 100,000 × g fraction, it was functionally similar to clusters identified in earlier fractions, suggesting no significant reorganization of the network.

A similar pattern, involving different metabolic processes, was observed in *C. auris*. Networks reconstructed from the five fractions showed comparable clustering profiles and conserved functional modules across ultracentrifugation speeds. Although minor variations in node density were detectable, the overall interaction signatures remained consistent among the 20,000 × g through 100,000 × g fractions, with a predominance of processes related to carbon metabolism and ribosome structure. In contrast, *C. parapsilosis* displayed a distinct pattern. The 20,000 × g and 40,000 × g fractions generated highly similar interaction networks, sharing comparable functional clusters and network architecture highly focused on translational processes. However, the 60,000 × g, 80,000 × g, and 100,000 × g fractions formed networks with a different organizational profile, indicating a shift in predicted interaction signatures at higher centrifugation speeds. Together, these analyses indicate that predicted protein interaction signatures are largely conserved across fractions in *C. deuterogattii* and *C. auris*, whereas *C. parapsilosis* exhibits a clear separation between low-speed and higher-speed ultracentrifugation fractions.

## Discussion

The need for improved methodologies to analyze fungal EVs has been consistently emphasized in the current literature (Rodrigues et al. 2023, 2025; Rodrigues and Nimrichter 2022). Protocols that focus on improving the yield of EV samples (Reis et al. 2019), in conjunction with the separation of subpopulations (Piffer et al. 2024), optimized production in fungal cultures (Reis et al. 2021), and regulation of vesicle biogenesis, composition, and trafficking based on nutrient availability (Cleare et al. 2020; Kabani et al. 2020) have been proposed. Despite the progress made in identifying subpopulations and biomarkers (Dawson et al. 2020; McKenna et al. 2023), the understanding of whether fungal EVs are comparable to their mammalian counterparts in terms of biogenesis and physical-chemical properties remains limited. In the *Cryptococcus* model, EV classification based on biogenesis is particularly challenging, as the vast majority of vesicles contain GXM (Rizzo et al. 2021). This polysaccharide is synthesized intracellularly and secreted via post-Golgi vesicles (Yoneda and Doering 2006) that are expected to fuse with the plasma membrane and release their contents into the fungal periplasm, rather than being detected as intact cargo in the extracellular milieu. The enrichment of GXM within cryptococcal EVs therefore points to the involvement of noncanonical and still poorly understood mechanisms of vesicle biogenesis. Together, these observations not only reinforce the need for improved analytical approaches to study fungal EVs, but also highlight the still underappreciated complexity of these structures.

A central finding of this study is that fungal EV heterogeneity is not effectively resolved by sequential ultracentrifugation. Across all centrifugal forces tested, each fraction contained bona fide vesicles as demonstrated by transmission electron microscopy. In *Cryptococcus*, separating GXM-containing EVs from other vesicle populations is not straightforward, as indicated by two independent studies using the same ultracentrifugation-based iodixanol gradient. Using this approach, Reis and colleagues reported that GXM accumulates in fractions 7 and 8 of twelve gradient fractions (Reis et al. 2021). Piffer and co-workers found that most cryptococcal EVs also concentrate in these two fractions (Piffer et al. 2024). Together, these observations support the idea that most cryptococcal EVs are associated with GXM (Rizzo et al. 2021), which complicates the analysis of polysaccharide-free EVs. Our initial hypothesis that GXM content would influence EV sedimentation behavior was contradicted by our data. All fractions were equally competent in transferring polysaccharide to acapsular cells. In parallel, we found no consistent relationship between vesicle size, GXM content, and sedimentation speed. In the three pathogens investigated in this study, EV populations displayed comparable size distributions across fractions in all three species analyzed. Together, these findings indicate that size and cargo density do not adequately explain the behavior of fungal EVs under differential ultracentrifugation. Importantly, our experimental approach focused on EV-associated polysaccharides. It remains unknown how the widely reported heterogeneity of extracellular GXM (Frases et al. 2008) affects the EV cargo and, additionally, patterns of polysaccharide incorporation by cryptococcal cells.

Our observations challenge a widely held assumption derived from mammalian EV studies, in which sedimentation profiles are often interpreted as proxies for vesicle subtype based on biophysical properties (Morris et al. 2024; Rupert et al. 2017). In contrast, our results suggest that fungal EV sedimentation is governed by more complex and potentially species-specific physicochemical features, possibly including membrane composition, surface properties, and aggregation tendencies. Indeed, aggregate formation could explain the observed differential sedimentation profiles despite similar compositions, as larger particles composed of similar vesicles would exhibit increased effective density while retaining comparable molecular components. These factors may override simple density- or size-based predictions and help explain why bona fide EVs are recoverable across a broad range of centrifugal forces. Moreover, differential ultracentrifugation, at least within the range commonly used in the field, did not segregate fungal EVs into clearly distinct subpopulations. Instead, the data support a model in which fungal EVs form a continuum of overlapping populations that co-sediment across a broad range of centrifugal forces. Our results suggest that classifications of EVs as “exosome-like” or “microvesicle-like”, when applied to fungi, may largely reflect methodological artifacts rather than true biological partitioning of vesicle subtypes.

In contrast to the limited discriminatory power of ultracentrifugation within species, we observed differences in sedimentation behavior across species. EVs from *C. deuterogattii* were predominantly recovered at higher centrifugal forces, whereas those from *Candida* species were enriched in lower-speed fractions. Notably, *C. parapsilosis* displayed a clear partitioning pattern, with low-speed fractions clustering separately from higher-speed fractions at the proteomic level. These findings indicate that EV sedimentation in these fungal species is not governed by universal principles but instead represents an intrinsic, species-dependent trait. Such differences are likely rooted in fundamental aspects of fungal biology, including variations in vesicle surface composition, and the mechanisms underlying EV biogenesis and release (Morris et al. 2024; Rupert et al. 2017). Our main findings are summarized in Table 1, and collectively these observations argue strongly against the use of standardized, one-size-fits-all isolation protocols in fungal EV research. Instead, they highlight the need for species-specific methodological optimization and caution against direct comparisons of EV datasets generated under identical centrifugation schemes across phylogenetically and biologically distinct fungi. Notably, our study was conducted on single strains of three distinct fungal species, and the diversity of EVs has been demonstrated at least within the *Cryptococcus* genus (Reis et al. 2021). This observation suggests that the properties herein described necessitate validation in additional fungal species and strains, including the various clades of *C. auris*.

**Table 1:** Comparison between expected ultracentrifugation model and observed fungal EV behavior across species

Aspect	Expected model based on conventional EV biology	Observed findings in this study	Species dependent pattern
Relationship between density or cargo and sedimentation	EVs with higher density or heavier cargo sediment at lower speeds	No correlation between GXM content and sedimentation across fractions	Specific for *C. deuterogattii*
EV recovery across ultracentrifugation speeds	Distinct EV subpopulations enriched at specific speeds	Bona fide EVs recovered across entire range from 20,000 to 100,000 g	Generalizable across all three species
Vesicle size versus sedimentation	Larger vesicles expected at lower speeds, smaller at higher speeds	No consistent size based segregation across fractions	Minor species specific variations but no systematic pattern
Proteomic composition across fractions	Distinct subpopulations expected to show distinct proteomes	High overlap of proteins across all fractions within each species	Degree of variability differs among species
Quantitative protein differences	Marked enrichment of subsets in specific fractions expected	Limited quantitative differences within species	More pronounced differences in *Candida* compared to *Cryptococcus*
EV subpopulation separation by ultracentrifugation	Method expected to separate biologically distinct EV classes	No clear separation, fractions represent overlapping populations	Clear separation only in *C. parapsilosis* at interaction network level
EV aggregation	Not a central parameter in classical models	Aggregates observed across all fractions and may influence sedimentation	Present in all species without clear fraction enrichment
Implication for methodology	Standard ultracentrifugation protocols broadly applicable	Fixed protocols may be misleading for fungal EV studies	Strong need for species specific optimization

More broadly, our results prompt a re-evaluation of the conceptual framework used to define EV subpopulations in fungi. The widely used classification into “exosome-like” and “microvesicle-like” particles, largely inherited from mammalian EV biology, implies the existence of discrete vesicle classes that can be separated based on physical properties such as size and sedimentation behavior. We therefore propose that fungal EV classification should move away from operational definitions based on isolation procedures and instead incorporate criteria grounded in fungal biology, including molecular cargo and functional properties. Developing such a framework will be critical for advancing the field and for enabling more meaningful comparisons across studies and species.

## Methods

### Strains and Media

The fungal strains used in this study were *Cryptococcus deuterogattii* R265, *Cryptococcus deneoformans* Cap67Δ, *Cryptococcus neoformans* H99, *Candida auris* MMC2, and *Candida parapsilosis* GA-1. Strains were stored in 16% glycerol at − 80 °C. For culture propagation, the different strains were incubated in liquid Yeast Peptone Dextrose (YPD) medium with agitation at 200 rpm at 30 °C for 24 h. All phosphate-buffered saline (PBS) solutions used in the experiments were filtered through membranes with 0.22 μm pores. For blocking steps and antibody dilutions, PBS containing 1% bovine serum albumin (PBS-BSA) was used.

### EV Isolation

Fungal cultivation followed by EV isolation was based on a protocol described by our group based on growth of fungal cells on solid medium (Reis et al. 2019). Cells were counted and adjusted to a density of 5.25 × 10⁷ cells/mL in 200 µL of YPD. These cell suspensions were spread onto YPD agar plates (90 mm × 15 mm Petri dishes containing 25 mL of medium) and incubated for 24 h at 30 °C until confluence was reached. Three (EV incorporation), forty (total proteomics), or sixty (fraction proteomics) plates were used for EV isolation, depending on the experiment. Cells were recovered from the plates using inoculation loops or cell scrapers. Recovered cells were transferred to centrifuge tubes containing 30 to 150 mL of PBS, depending on the number of plates scrapped. A 5 µL aliquot of the cell suspension was collected for cell counting in a Neubauer chamber to estimate the total number of recovered cells. To obtain a cell-free supernatants, the samples were first centrifuged at 5,000 ×g for 15 min at 4 °C. The supernatants were collected and centrifuged again at 15,000 ×g for 15 min at 4 °C to remove cellular debris. The resulting supernatants were filtered through membranes with 0.45 μm pores and sequentially centrifuged at 20,000, 40,000, 60,000, 80,000, and 100,000 ×g for 1 h at 4 °C. After each centrifugation step, before proceeding to the next higher speed, the supernatants were transferred to new ultracentrifuge tubes, and the pellets at the bottom of the tubes were resuspended in 150 to 300 µL of PBS. EV preparations were stored at − 20 °C until further use.

### Transmission Electron Microscopy

EV samples were placed over nickel grids (01753 N, PELCO^®^) and incubated for 1 h. The grids were washed once with PBS and dried with filter paper. For fixation, the grids were placed in Karnovsky solution (5% glutaraldehyde and 4% paraformaldehyde in 18 MΩ water, pH 7.4) for 10 min. The grids were then washed three times in 0.1 M sodium cacodylate buffer and dried again with filter paper. The samples were negatively stained with 5% uranyl acetate for 2 min, washed once with water, and dried. Images were acquired using a JEOL 1400Plus transmission electron microscope operating at 100 kV. Diameters were determined in 150 particles per sample using Fiji (Schindelin et al. 2012).

### Nanoparticle Tracking Analysis (NTA)

Samples were diluted in PBS (50–100,000-fold) to reach the optimal particle concentration range from 3 × 10⁷ to 2 × 10⁹ particles/mL. Analyses were performed using a NanoSight LM10 nanoparticle analysis system (Malvern Panalytical, Malvern, UK) with continuous sample injection (syringe pump flow rate of 50) (Reis et al. 2019). Particles were tracked using a scientific complementary metal–oxide–semiconductor (CMOS) camera by light scattering from a 488 nm laser. The camera level was set between 14 and 16, and the detection threshold between 3 and 7. Each sample was recorded three times for 60 s. Data were analyzed using NTA 3.0 software (Malvern Panalytical Ltd.).

### GXM Detection by Enzyme-Linked Immunosorbent Assay (ELISA)

Sample normalization was determined according to protein content, obtained by Pierce™ Micro BCA Protein Assay Kit (Thermo Scientific™, catalog number 23235), in accordance with manufacturer’s guidelines. The equivalent to 6 µg of protein were collected in 90 µL of PBS, followed by vacuum centrifugation to dryness. Alternatively, 10^10^ EV particles were processed similarly. To disrupt the EVs, 5 µL of absolute methanol and 45 µL of chloroform were added, followed by vortexing, with immediate precipitation of GXM. The microtubes were then left open on the laboratory bench overnight to allow complete solvent evaporation. The resulting material was suspended in 180 µL of PBS and 75 µL used to coat ELISA plates (Rodrigues et al. 2007). GXM was detected with the monoclonal antibody 18B7, kindly provided by Arturo Casadevall (Johns Hopkins University, Baltimore), as previously described (Casadevall et al. 1992). Colorimetric reactions were measured spectrophotometrically at 405 nm using a Synergy H1M2F microplate reader.

### Coating of Cryptococcal Acapsular with GXM Obtained from EVs

In microtubes, 2 × 10⁶ cells of the Cap67 strain of *C. deneoformans* were suspended in 200 µL of PBS. To each suspension, 5.4 × 10⁸ EV particles from each centrifugation speed were added. The systems were incubated for 24 h at 37 °C in a 5% CO₂ atmosphere. The cells were washed three times with PBS and fixed in 100 µL of 4% paraformaldehyde in 0.1 M sodium cacodylate buffer for 20 min at room temperature. The cells were again washed in PBS and blocked with 500 µL of PBS-BSA for 1 h at room temperature. After washing, 200 µL of antibody 18B7 (10 µg/mL) was added followed by incubation for 1 h at room temperature. The cells were washed and incubated for an additional 1 h at room temperature with 200 µL of an anti-mouse immunoglobulin antibody (Alexa Fluor 546-conjugated) at 3.33 µg/mL. After washing, the cells were resuspended in 100 µL of PBS. From this suspension, 5 µL was placed onto glass microscope slides, covered with coverslips, and sealed with colorless nail polish for fluorescence microscopy. Alternatively, the cells were analyzed by flow cytometry. For cytometer setup (FACSCanto™ II), two controls were included: mutant cells without staining, and wild-type H99 cells stained for GXM.

### Preparation of EV samples for proteomics

EVs from *C. deuterogattii* R265, *C. auris* MMC2 and *C. parapsilosis* GA1 were fractionated by sequential centrifugation as described before in this section to generate biological triplicates. Samples were lysed with 0.1 mm zirconium beads (1:1, v/v) followed by five cycles of agitation (1 min each), interspersed with 1-min cooling intervals, using the bead beater L-BEADER (Loccus). Subsequently, 8 M urea was added to the pellet, and the lysis process was repeated for five cycles under the same conditions. The lysates were centrifuged at 18,000 × g at 4 °C for 15 min, supernatants were collected, and protein concentrations were determined by fluorometric quantification using a Qubit fluorometer (Thermo Fisher Scientific Inc.). Samples containing 100 µg of protein were reduced with dithiothreitol (DTT; final concentration 10 mM) for 30 min at 56 °C. Samples were cooled to room temperature and alkylated with 15 mM iodoacetamide for 25 min in the dark. The solution was then incubated again with 10 mM DTT for 15 min at room temperature in the dark. Protein samples were diluted with 50 mM ammonium bicarbonate (ABC) to reduce the urea concentration to 2 M, and pH was adjusted to 7–8. Samples were then enzymatically digested with trypsin (1:50, w/w) and incubated overnight at 37 °C. Following digestion, trifluoroacetic acid (TFA) was added to a final concentration of 0.5% and the digested proteins were quantified again with the Qubit system (Thermo Fisher Scientific Inc.). Peptides were desalted and concentrated using StageTip columns with C18 Empore disks (3 M) inserted into 200-µL pipette tips. The columns were activated with 100 µL of 100% methanol and centrifuged for 2 min at 1,000 × g followed by equilibration with 100 µL of 0.1% formic acid under the same centrifugation conditions. Next, 200 µL of each sample was loaded onto the column and centrifuged until complete passage. Finally, the columns were washed twice with 200 µL of 0.1% formic acid.

### Mass Spectrometry

The analysis was performed using an Ultimate 3000 nanoLC system (Thermo Fisher Scientific Inc.) and all mass spectrometry proteomics data have been deposited to the ProteomeXchange Consortium via the PRIDE (Perez-Riverol et al. 2022) partner repository with the dataset identifier PXD076414. Previously desalted peptides were analyzed in technical duplicates and loaded onto reverse-phase columns (30 cm length, 75 μm inner diameter) packed with ReproSil-Pur C18-AQ resin (Dr. Maisch; 3 μm particles; 120 Å pore size). Peptides were separated using a 120-min gradient, ranging from 5% to 40% mobile phase B (95% acetonitrile with 0.1% formic acid). The eluate was directly introduced into an Orbitrap Fusion Lumos mass spectrometer (Thermo Fisher Scientific Inc.). Mass spectra were acquired on the Orbitrap Fusion Lumos in data-dependent acquisition (DDA) mode, automatically alternating between full MS and MS/MS scans, with a dynamic exclusion of 45 s. Full MS scans were acquired at a resolution of 60,000 (at m/z 200), with a cycle time of 2 s. The most intense ions with charge states of 2 + and 3 + were isolated and fragmented by higher-energy collisional dissociation (HCD) using a normalized collision energy of 30%. The chromatographic gradient and instrument operations were controlled using Xcalibur 4 software (Thermo Fisher Scientific Inc.). Parameters included a spray voltage of 2.6 kV, a capillary temperature of 250 °C, no auxiliary or sheath gas flow, and automatic gain control (AGC) enabled. The S-lens RF level was set to 70%.

### Peptide Spectral Matching (PSM) and PSM Validation

Peptide spectral matching (PSM) and subsequent validation were performed using modules from the PatternLab for Proteomics V software suite (Santos et al. 2022) (http://www.patternlabforproteomics.org/), employing the multiplexed spectra identification feature based on the YADA 3.0 deconvolution algorithm; this allows for the identification of more than one peptide per mass spectrum (Clasen et al. 2022, 2023). A target–decoy database was constructed to ensure reliable identifications and consisted of *C. deuterogattii* R265. The database was retrieved from FungiDB (https://fungidb.org/fungidb/app) on October 10, 2025, corresponding to *C. gattii* VGII R265. For *C. auris* MMC2 (proteome ID: UP000230249) and *C. parapsilosis* GA1 (proteome ID: UP000005221), the protein datasets were obtained from UniProt (http://www.uniprot.org/) on October 25, 2025. These databases included the reversed sequence of each entry, as well as 127 common mass spectrometry contaminants. Tandem mass spectra were searched against this database using the Comet search engine (Eng et al. 2015). Searches were restricted to tryptic peptides allowing up to two missed cleavages, with carbamidomethylation of cysteine defined as a fixed modification and oxidation of methionine as a variable modification. PSM validation was carried out using the Search Engine Processor (SEPro) (Carvalho et al. 2012). Identifications were grouped according to charge state (+ 2 and ≥ + 3), and Comet-derived parameters, including cross-correlation (XCorr), DeltaCN, and spectrum count score, were combined to generate a Bayesian discriminator. PSMs were ranked based on discriminator values, and cutoff thresholds were established to achieve a 1% false discovery rate (FDR) at the protein level, as estimated by the number of labeled decoy hits (Barboza et al. 2011). Only peptide sequences containing six or more amino acid residues were considered. Additional post-processing filters were applied to retain PSMs with a mass error below 6 ppm, a minimum XCorr value of 2.0, and proteins supported by at least one independent piece of evidence. These criteria ensured that the final dataset met a global protein-level FDR of 1%.

### Relative Protein Quantification, Protein-protein Interactions and Functional Classification. Protein Quantification was Performed using Extracted ion Chromatograms (XIC) (Neilson et al. 2011)

Only proteins identified with at least two unique peptides were retained, and contaminants and decoys entries were removed. The data were initially analyzed with modules provided by PatternLab for Proteomics V: Overlap Analysis (Venn-Diagram) and T-Fold (Protein abundance)(Carvalho et al. 2016), both filtered to include results from at least two biological replicates. Functional enrichment, based on biological process gene ontology terms, of exclusive *Candida sp.* proteins was performed using PANTHER classification system (available at https://pantherdb.org/geneListAnalysis.do) on February 2, 2026. Additional protein–protein interaction analyses were performed using STRING software (available at https://string-db.org/ ) 28 on January 10, 2026.

### Statistical Analysis

GXM content in fractions obtained at different centrifugation speeds was quantified by ELISA using two technical replicates from each of two independent experiments. Values were independently normalized by total protein concentration and by particle number determined by NTA. For each normalization approach, differences among centrifugation speeds were assessed by analysis of variance (ANOVA), with independent experiment included as a blocking factor. Similarly, for the automatic diameter measurements, the mean vesicle diameter for fractions obtained at each ultracentrifugation speed was calculated from three technical replicates across three independent experiments. Differences among speeds were evaluated by ANOVA, with independent experiment included as a blocking factor. For both analyses, the mean value from each independent experiment was used for graphical representation. In addition, for vesicle diameter comparison at the individual-particle level, sixty vesicles were randomly selected from each fraction obtained at different ultracentrifugation speeds within a single experimental replicate. The distributions of diameter values at each speed were compared using non-parametric methods. Overall differences were assessed using the Kruskal-Wallis test, followed by Dunn’s post-hoc test with Holm adjustment for multiple comparisons. Data were presented in boxplots with individual observations, and the diameter axis was presented on a base-10 logarithmic scale. For all analyses, differences with *p* < 0.05 were considered statistically significant.

## Supplementary Information

Below is the link to the electronic supplementary material.

Supplementary Material 1

Supplementary Material 2

Supplementary Material 3

Supplementary Material 4

Supplementary Material 5

Supplementary Material 6

## Data Availability

Reviewer access detailsLog in to the PRIDE website using the following details: Project accession: PXD076414Token: qbgvU7Qp3szXAlternatively, reviewer can access the dataset by logging in to the PRIDE website using the following account details: Username: reviewer_pxd076414@ebi.ac.ukPassword: To7lU23pHpVx.

## Declarations

### Generative AI and AI-assisted technologies in the writing process

During manuscript preparation, L.J.J. used ChatGPT (chat.openai.com) exclusively for grammar correction. The revised text was subsequently reviewed by M.L.R. and then by all coauthors. We collectively take full responsibility for the content of the manuscript in its current form.
